# Developmental Timing Determines the Protective Effect of Maternal Electroacupuncture on Perinatal Nicotine Exposure-Induced Offspring Lung Phenotype

**DOI:** 10.1155/2020/8030972

**Published:** 2020-02-27

**Authors:** Jian Dai, Bo Ji, Guozhen Zhao, Yawen Lu, Yitian Liu, Qiujie Mou, Reiko Sakurai, Yana Xie, Qin Zhang, Shuang Xu, Virender K. Rehan

**Affiliations:** ^1^School of Acupuncture-Moxibustion and Tuina, Beijing University of Chinese Medicine, Beijing 100029, China; ^2^Department of Pediatrics, Lundquist Institute for Biomedical Innovation at Harbor-UCLA Medical Center, David Geffen School of Medicine at UCLA, Los Angeles, CA 90502, USA

## Abstract

*Introduction*. Environmental exposure of the developing offspring to cigarette smoke or nicotine is an important predisposing factor for many chronic respiratory conditions, such as asthma, emphysema, pulmonary fibrosis, and so forth, in the exposed offspring. Studies showed that electroacupuncture (EA) applied to maternal “Zusanli” (ST36) acupoints during pregnancy and lactation protects against perinatal nicotine exposure- (PNE-) induced lung damage. However, the most effective time period, that is, prenatal vs. postnatal, to attain this effect has not been determined.

**Objective:**

To determine the most effective developmental timing of EA's protective effect against PNE-induced lung phenotype in the exposed offspring.

**Methods:**

Pregnant rats were given (1) saline (“S” group); (2) nicotine (“N” group); (3) nicotine + EA, exclusively prenatally (“Pre-EA” group); (4) nicotine + EA, exclusively postnatally (“Post-EA,” group); and (5) nicotine + EA, administered both prenatally and postnatally (“Pre- and Post-EA” group). Nicotine was injected once daily (1 mg/kg, 100 *μ*l) and EA was administered to bilateral ST36 acupoints once daily during the specified time-periods. At the end of the experimental periods, key hypothalamic pituitary adrenal (HPA) axis markers in pups and dams, and lung function, morphometry, and the central molecular markers of lung development in the offspring were determined.

**Results:**

After nicotine exposure, alveolar mean linear intercept (MLI) increased, but mean alveolar number (MAN) decreased and lung PPAR*γ* level decreased, but glucocorticoid receptor (GR) and serum corticosterone (Cort) levels increased, in line with the known PNE-induced lung phenotype. In the nicotine exposed group, maternal hypothalamic corticotropin releasing hormone (CRH) level decreased, but pituitary adrenocorticotropic hormone (ACTH) and serum Cort levels increased. In the “Pre- and Post-EA” groups, PNE-induced alterations in lung morphometry, lung development markers, and HPA axis were blocked. In the “Pre-EA” group, PNE-induced changes in lung morphometry, GR, and maternal HPA axis improved; lung PPAR*γ* level decreased, but glucocorticoid receptor (GR) and serum corticosterone (Cort) levels increased, in line with the known PNE-induced lung phenotype. In the nicotine exposed group, maternal hypothalamic corticotropin releasing hormone (CRH) level decreased, but pituitary adrenocorticotropic hormone (ACTH) and serum Cort levels increased. In the “Pre- and Post-EA” groups, PNE-induced alterations in lung morphometry, lung development markers, and HPA axis were blocked. In the “Pre-EA” group, PNE-induced changes in lung morphometry, GR, and maternal HPA axis improved; lung PPAR

**Conclusions:**

Maternal EA applied to ST36 acupoints during both pre- and postnatal periods preserves offspring lung structure and function despite perinatal exposure to nicotine. EA applied during the “prenatal period” affords only limited benefits, whereas EA applied during the “postnatal period” is ineffective, suggesting that the EA's effects in modulating PNE-induced lung phenotype are limited to specific time-periods during lung development.

## 1. Introduction

Despite well-established dangers of tobacco to human health, exposure of pregnant women to mainstream or sidestream smoke remains extremely high [1]. Although among the high-income women, the number of smokers is decreasing, among low-income women, this number is increasing [2]. Importantly, over half of the smokers continue to smoke while pregnant [3]. Considerable evidence supports that nicotine is the main harmful substance in cigarettes, which rapidly crosses the placenta and accumulates in the fetus in concentrations much higher than maternal serum concentrations [4]. Prenatal exposure to nicotine not only affects the survival and birth weight of infants [5, 6], but also adversely affects many developing systems including but not limited to the nervous, circulatory, immune, and respiratory systems [7–10]. Its effects are especially pronounced on the developing lung [11], as it predisposes the exposed offspring to many chronic respiratory conditions such as asthma, emphysema, pulmonary fibrosis, and so forth. [12–15]. These effects appear to be permanent, lasting to adulthood and some can even be potentially transmitted to future generations [16, 17].

Nicotine's effects on the developing lung have been largely attributed to a disruption in epithelial-mesenchymal paracrine signaling, the central component of which is the nuclear transcription factor peroxisome proliferator-activated receptor-*γ* (PPAR*γ*). PPAR*γ* is centrally involved in alveolar and airway development [18–20] and is a key determinant of the alveolar fibroblast differentiation to lipofibroblasts, which are essential for alveolar development, homeostasis, and injury repair [18, 21]. Lung-specific PPAR*γ* knockout mice show enlarged alveolar sacs, increased apoptotic cells, and an enlarged lung volume, highlighting PPAR*γ*'s indispensable role in lung development [19, 20]. Nicotine, by down-regulating PPAR*γ*, drives alveolar lipofibroblasts to transdifferentiate to myofibroblasts, which are the hallmarks of all chronic lung conditions including the perinatal nicotine exposure- (PNE-) induced lung damage [22, 23]. Supporting these observations, in experimental animal models, blocking lipofibroblast-to-myofibroblast differentiation, using PPAR*γ* agonists blocks and/or reverses the PNE-induced lung damage in the exposed offspring [24, 25].

Hypothalamic pituitary adrenal (HPA) axis, by regulating the production of glucocorticoids, also performs an essential role in lung development and maturation [26]. Glucocorticoids act on the glucocorticoid receptor (GR), expressed in the developing lung, stimulating alveolar epithelial-mesenchymal cross-talk, and increase surfactant production. However, excessive glucocorticoids, either endogenous or administered exogenously, can hinder lung development, predisposing to conditions such as childhood asthma [27] and emphysema [28]. Evidence suggests that perinatal nicotine exposure disrupts maternal and offspring HPA axes, increasing maternal and offspring serum corticosterone (Cort) levels, which impacts offspring growth and development negatively [29–32]. Thereby, PNE-induced lung damage, at least, in part, can be attributed to altered maternal and offspring HPA axes.

Currently, there is no clinically safe and effective pharmacologic intervention to prevent or treat PNE-induced lung damage [25, 33–39]. Interestingly, electroacupuncture (EA) is known to treat a number of respiratory conditions, such as allergic asthma and acute lung injury [40, 41]. By regulating HPA axis, EA also improves airway inflammation associated with asthma [42]. More importantly, experimentally, we have recently shown that EA applied to maternal “Zusanli” (ST36) acupoints during pregnancy and lactation (from embryonic day 6 [E6] to postnatal day 21 [PND21]) protects against PNE-induced lung damage [31, 32]. However, the most effective time-period, that is, prenatal vs. postnatal, to attain this effect has not been determined. Since lung morphogenesis is a complex, finely orchestrated program with specific signaling pathways involved at specific stages during development, we hypothesize that the EA's effect in modulating PNE-induced lung phenotype is limited to specific time-periods during lung development. Here we compare EA's protective effect against nicotine-induced lung phenotype, when it is administered exclusively “prenatally” (embryonic, pseudoglandular, canalicular, and early saccular stages of lung development), exclusively “postnatally” (late saccular and alveolar stages of lung development), or both “pre- and postnatally” (all stages of lung development).

## 2. Materials and Methods

### 2.1. Animals

Approval was obtained from the Beijing University of Chinese Medicine experimental animal Ethics Committee in 2017 and all animal procedures were performed in accordance with the “Guide to the Care and Use of Experimental Animals” of the China Animal Welfare Commission. Thirty female and ten male speciﬁc pathogen-free Sprague-Dawley rats (11 weeks old) without prior mating history were obtained (SPF, Beijing, Biotechnology Co., Ltd., production license number: SCXK (Beijing) 2006-0002). Animals were housed at a constant temperature and humidity environment with 12 hours of alternate light and dark cycle, with the provision of ad lib food and water. The feeding cages and water bottles were regularly disinfected.

### 2.2. Experimental Protocol

In line with a well-established model [31, 32], saline or nicotine injections (saline: 100 *μ*l volume once daily and nicotine: 1 mg/kg in 100 *μ*l volume once daily) were started on E6, and continued throughout pregnancy and lactation, that is, up to PND21 (except on the day of delivery). The saline group (“S” group) was injected saline once daily. The nicotine group (“N” group) was injected nicotine once daily. For the prenatal EA group (“Pre-EA group”), nicotine injection was the same as in the “N” group, but these dams were also administered EA to bilateral ST36 acupoints from E6 to the day of delivery. For the postnatal EA group (“Post-EA” group), nicotine injection was the same as in the “N” group, but these animals were administered EA to bilateral ST36 acupoints from PND1 to PND21. The prenatal and postnatal EA group (“Pre- and Post-EA” group) was administered nicotine similar to the “N” group, but these animals also received EA at bilateral ST36 acupoints from E6 to PND21 (except on the day of delivery). On PND21, pulmonary function testing was performed before sacrificing pups for lung tissue and serum collection and dams for the hypothalamus, pituitary, and serum collection.

### 2.3. Electroacupuncture Protocol

The ST36 acupoints were identified at the posterolateral side of knee-joint about 5 mm below the head of the fibula, as detailed in “Experimental Acupuncture Science” [43]. Disposable sterile acupuncture needles (0.20 mm × 13 mm, Beijing Hanyi Medical Instruments Centre, China) were pierced to a depth of ∼0.7 cm at bilateral ST36 acupoints (connecting to negative pole) and horizontally to a depth of ∼0.2 cm into the skin below ST36 (connecting to positive pole). The EA parameters were, frequency 2/15 Hz; intensity 1 mA; and duration 20 minutes, administered once a day. For consistency, acupuncture was performed by the same operator between 10 a.m. to twelve noon throughout the study period.

### 2.4. Pulmonary Function Testing

Pulmonary function testing was performed by the Respiratory Function Instrument with Buxco FinePointe software (Buxco, USA). The pups were intraperitoneally injected with 2% pentobarbital (5.5 mg/100g) for anesthesia, tracheotomized, cannulated, and connected to a ventilator for plethysmography. After a period of steady breaths, the lung resistance (RL), dynamic compliance (Cdyn), minute ventilation volume (MV), and peak expiratory flow (PEF) were recorded.

### 2.5. Lung Morphology

At sacrifice, pup lungs were fully inflated with 4% paraformaldehyde (PFA) in PBS with constant pressure; after ligation, the lungs were submerged in 4% PFA for about 5 h, followed by immersion in 30% sucrose in PBS. The left lung was used for paraffin embedding, cut into 5 *μ*m slices, which for lung morphometry were stained with hematoxylin and eosin (H&E). Subsequently, lung tissue morphology was assessed by determining mean linear intercepts (MLI) and mean alveolar numbers (MAN) using previously described methods [44].

### 2.6. Offspring Lung PPAR*γ* and Glucocorticoid Receptor and Maternal Hypothalamic Corticotropic Releasing Hormone and Pituitary Adrenocorticotropic Hormone ELISA

Offspring lung tissue and maternal hypothalamic and pituitary tissues were homogenized and the supernatants were collected for detecting PPAR*γ* (Cusabio, China, Catalog#: CSBE08624r), GR (Cusabio, China, Catalog#: CSB-E08747r), CRH (Immunoway, USA, Catalog#: KE1318), and ACTH (Raybiotech, USA, Catalog#: EIAR-ACTH 0524197055) using ELISA as per manufacturer's instructions.

### 2.7. Radioimmunoassay for Serum Corticosterone Levels in Offspring and Mother

Serum Cort levels in the mother and offspring rats were performed using radioimmunoassay as manufacturer's instructions (BioSino Bio-Technology and Science Inc. Catalog#: HY-068B).

### 2.8. Offspring Lung PPAR*γ* mRNA Expression by Real-Time PCR

The method for RNA extraction, Real-time PCR, and the primers information of PPAR*γ* and GAPDH have been described previously [31].

### 2.9. Statistical Analysis

The data are expressed as mean ± SD. Statistical analysis was performed using SPSS statistical software (SPSS Inc., USA). One-Way ANOVA-Bonferroni test was used for the comparison of differences between groups, and *P* < 0.05 was considered statistically significant.

## 3. Results

### 3.1. Effect of Maternal EA during Different Developmental Time-Periods on PNE-Induced Changes in Offspring Lung Function

**Figure 1:**
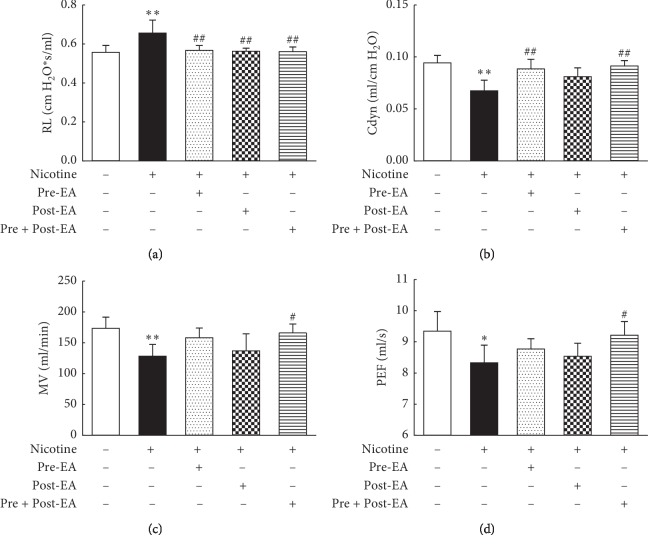
Effect of maternal EA during different developmental time-periods on PNE-induced changes in offspring pulmonary function. (a) RL. (b) Cdyn. (c) MV. (d) PEF. Values are mean ± SD; *n* = 6 per group. ^*∗*^*P* < 0.05, ^*∗∗*^*P* < 0.01 vs. control; ^#^*P* < 0.05, ^##^*P* < 0.01 vs. nicotine.

Compared with the “S” group, in the “N” group, Cdyn, MV, and PEF decreased (*P* < 0.01, <0.01, and <0.05, respectively), while RL increased (*P* < 0.01) significantly. Compared with the “N” group, in the “Pre-EA” group, Cdyn increased (*P* < 0.01) and RL decreased (*P* < 0.01) significantly; however, the MV and PEF were not different (*P* > 0.05). Compared with the “N” group, the RL decreased significantly (*P* < 0.01) in the “Post-EA” group; however, the Cdyn, MV, and PEF were not significantly different (*P* > 0.05). Furthermore, the Cdyn, MV, and PEF increased (*P* < 0.01, <0.05, and <0.05, respectively), while RL decreased (*P* < 0.01) significantly, in “Pre- and Post-EA” group vs. the “N” group (Figure 1).

### 3.2. Effect of Maternal EA during Different Developmental Time-Periods on PNE-Induced Changes in Offspring Lung Morphometry

**Figure 2:**
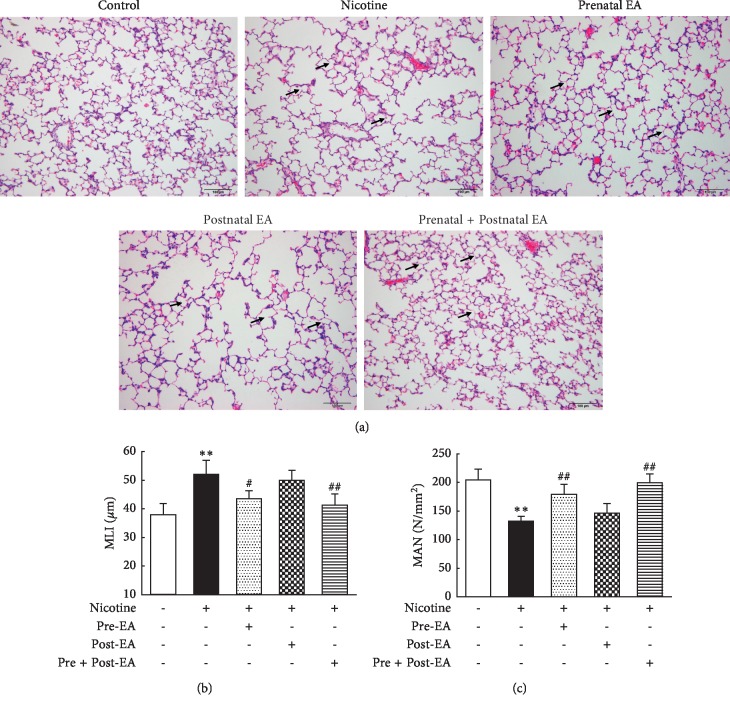
Effect of maternal EA during different developmental time-periods on PNE-induced changes in offspring lung morphometry. (a) Representative H&E-stained lung sections. Magnification ×20; arrows point to the integrity and/or rupture of alveolar walls. (b) MLI. (c) MAN. Values are mean ± SD; *n* = 5 per group; ^*∗∗*^*P* < 0.01 vs. control; ^#^*P* < 0.05, ^##^*P* < 0.01 vs. nicotine.

The photomicrographs of the H&E-stained sections showed that the alveolar structure in group “S” was intact and the alveolar septum relatively complete. Compared with the “S” group, the alveolar volume in the “N” group was significantly larger, as determined by the greater MLI (*P* < 0.01), accompanying a lower MAN (*P* < 0.01), and in parts ruptured and fused alveolar walls. Compared with the “N” group, the “Pre-EA” and the “Pre- and Post-EA” group had smaller alveolar volumes (*P* < 0.05 and <0.01, respectively, vs. the “N” group) and more alveoli (*P* < 0.01 vs. the “N” group), the rupture and fusion of alveolar walls improved; however, the “Post-EA” group was not different from the “N” group (*P* > 0.05 vs. the “N” group) (Figure 2).

### 3.3. Effect of Maternal EA during Different Developmental Time-Periods on PNE-Induced Changes in Offspring Lung PPAR*γ* mRNA and Protein Levels

**Figure 3:**
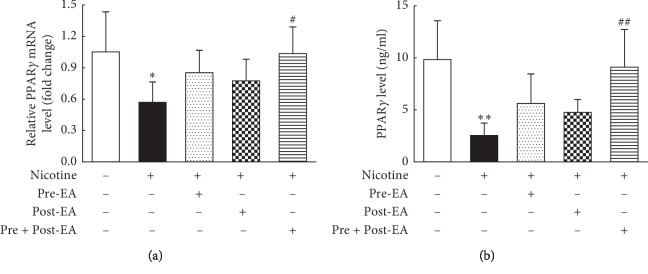
Effect of maternal EA during different developmental time-periods on PNE-induced changes in offspring lung PPAR*γ* mRNA and protein levels. (a) PPAR*γ* mRNA. (b) PPAR*γ* protein. Values are mean ± SD; *n* = 6 per group. ^*∗*^*P* < 0.05, ^*∗∗*^*P* < 0.01 vs. control; ^#^*P* < 0.05, ^##^*P* < 0.01 vs. nicotine.

Using Real-time PCR and ELISA, compared to the “S” group, PPAR*γ* mRNA (Figure 3(a)) and protein (Figure 3(b)) levels decreased significantly in the “N” group (*P* < 0.05, and <0.01, respectively). Both of these changes were blocked in the “Pre- and Post-EA” group (*P* < 0.05, and<0.01 vs. the “N” group); although the “Pre-EA” group showed a significant increase, it did not reach statistical significance (*P* > 0.05 vs. the “N” group); however, the “Post-EA” group was not different from the “N” group in both PPAR*γ* mRNA and protein levels (*P* > 0.05 for both).

### 3.4. Effect of Maternal EA during Different Developmental Time-Periods on PNE-Induced Changes in Offspring HPA Axis

**Figure 4:**
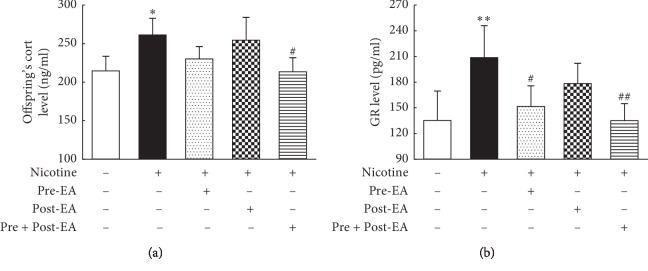
Effect of maternal EA during different developmental time-periods on PNE-induced changes in offspring. (a) Serum Cort of offspring. (b) Lung GR of offspring. Values are mean ± SD; *n* = 5-6 per group. ^*∗*^*P* < 0.05, ^*∗∗*^*P* < 0.01 vs. control; ^#^*P* < 0.05, ^##^*P* < 0.01 vs. nicotine.

The results showed that serum Cort (Figure 4(a)) and lung GR (Figure 4(b)) levels in the “N” group were significantly higher than in the “S” group (*P* < 0.05, and <0.01, respectively), which normalized in the “Pre- and Post-EA” group (*P* < 0.05, and <0.01 vs. the “N” group). Furthermore, in the “Pre-EA” group, compared with the “N” group, though the lung GR decreased significantly (*P* < 0.05), serum Cort was not significantly different (*P* > 0.05). Furthermore, the “Post-EA” group was not different from the “N” group in both (lung GR and serum Cort levels) of these parameters (*P* > 0.05).

### 3.5. Effect of Maternal EA during Different Developmental Time-Periods on PNE-Induced Changes in Maternal HPA Axis

**Figure 5:**
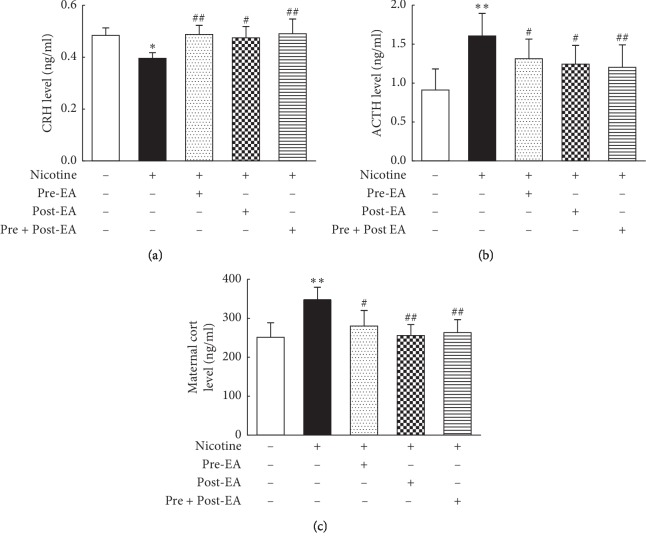
Effect of maternal EA during different developmental time-periods on PNE-induced changes in maternal HPA axes. (a) Maternal CRH. (b) Maternal ACTH. (c) Maternal Cort; Values are mean ± SD; *n* = 5-6 per group. ^*∗*^*P* < 0.05, ^*∗∗*^*P* < 0.01 vs. control; ^#^*P* < 0.05, ^##^*P* < 0.01 vs. nicotine.

The results showed that compared to the “S” group, the levels of maternal hypothalamic CRH decreased (*P* < 0.05, Figure 5(a)), while the pituitary ACTH (*P* < 0.01, Figure 5(b)) and serum Cort (*P* < 0.01, Figure 5(c)) levels increased significantly in “N” group. These changes were blocked in the “Pre-EA” (*P* < 0.01, <0.05, and <0.05, respectively), “Post-EA” (*P* < 0.05, <0.05, and<0.01, respectively), and “Pre- and Post-EA” (*P* < 0.01, <0.01, and <0.01, respectively) group.

## 4. Discussion

Exposure to mainstream or sidestream smoke during pregnancy is an important healthcare risk worldwide. It adversely affects offspring development, especially having a long-term detrimental effect on the respiratory health of the exposed offspring [45–47]. Considering nicotine's strong addictive effect and the extensive advertising by the tobacco companies to target teens, the problem of smoke exposure during pregnancy is unlikely to go away soon. Hence, finding novel, safe, and effective intervention strategies to mitigate the impact of perinatal tobacco exposure is of great public health significance.

Electroacupuncture is a modification of acupuncture that stimulates acupoints with low-frequency pulsed electrical current. Biologically, it is a combination of acupuncture stimulation and its consequent electrophysiological effects. As a nonpharmacologic therapy, EA is easy to operate and has minimal side effects [48]. ST36 is an acupoint of the “Stomach Meridian” and has been identified to be important for general improvement in health. It is effective in treating diseases of many organ systems, including the respiratory system [49, 50]. It also modulates HPA axis stability [51]. In general, the effects of acupuncture are determined by factors, such as the functional state of the body, stimulation parameters, acupoint selection, and the timing and duration of treatment. Regarding the timing of treatment, it was demonstrated that acupuncture treatment 4–7 days after the onset of facial paralysis is better than its administration either within the first 1–3 days or after 8–10 days of the onset of facial paralysis [52]. Similarly, for establishing a more efficient bladder control of a neurogenic urinary bladder following spinal cord injury, earlier intervention is better than later [53]. These studies indicate that the efficacy of acupuncture at different disease stages is different.

Mammalian lung morphogenesis is a complex, finely orchestrated program, which progresses through well defined, sequential stages to result in fully functional lung; for example, the rat lung development proceeds through the embryonic (E11–13), pseudoglandular (E13–18.5), canalicular (E18.5–20), saccular (E20-PND4), and alveolar (PND4–21) stages. Specific growth factors and signaling mechanisms regulate each stage and drive its progression to the next stage [54]. By comparing EA's protective effects against nicotine-induced lung phenotype, administered exclusively during the “prenatal period” (embryonic, psuedoglandular, canalicular, and early saccular stage of lung development), “postnatal period” (late saccular and alveolar stages of lung development), or both “prenatal and postnatal periods” (all stages of lung development), we found that the PNE-induced lung morphometric (MLI and MAN) and functional (Cdyn, PEF, MV, and RL) changes were effectively blocked only when EA was administered during both “prenatal and postnatal periods.” This is in line with our previous findings [31, 32]. However, its application exclusively during the “prenatal period” resulted in incomplete mitigation of perinatal nicotine-induced pulmonary functional changes, for example, nicotine's effects on Cdyn and RL were blocked, but not on PEF and MV. The application of EA exclusively during the “postnatal period” had even fewer effects; that is, it only blocked PNE-induced changes in RL but not in other pulmonary functional indices. These data suggest a graded efficacy of EA's beneficial effects when administered during both “prenatal and postnatal periods,” exclusively “prenatal period,” or exclusively “postnatal period,” with administration during both “pre- and postnatal periods” providing the maximum beneficial effect, while its administration exclusively during the “postnatal period” had the least beneficial effect.

PPAR*γ* is a ligand-activated transcription factor that plays a key role in regulating lipid storage and metabolism in various organs including the lung [55–57]. Experimentally, in a rat model, PNE down-regulated PPAR*γ* expression in the developing lung along with the associated nicotine-induced pulmonary structural and functional phenotype [25, 58]. EA applied to maternal ST36 acupoints during “pre- and postnatal periods” completely prevented the nicotine-induced decrease in pulmonary PPAR*γ* protein levels, in conjunction with blockage of the perinatal nicotine-induced pulmonary structural and functional changes. Interestingly, EA applied exclusively during the “prenatal period” only slightly blocked the PNE-induced decrease in pulmonary PPAR*γ* protein levels, which, not surprisingly, was accompanied by incomplete protection against PNE-induced pulmonary effects; that is, although the lung morphology improved, it only partially blocked nicotine's effects on pulmonary function. In contrast, administration of EA exclusively during the “postnatal period,” neither improved pulmonary PPAR*γ* protein levels nor nicotine's effects on lung structure and function.

To understand the mechanism of EA's effects on nicotine-induced pulmonary morbidity in the developing lung, it is important to understand nicotine's effects on maternal and fetal HPA axes and how these are affected by EA. Glucocorticoids are key players in mediating stress response on the HPA axis, both before and after birth [59, 60]. In general, maternal and fetal/neonatal glucocorticoid levels correlate closely. High maternal glucocorticoid levels can result in high blood circulatory levels in the fetus and infant through the placenta and breast milk, respectively [61, 62]. Nicotine increases glucocorticoid synthesis in maternal adrenals, decreases placental 11*β*-HSD-2 activity, and compromises the placental barrier to maternal glucocorticoids, which leads to fetal overexposure to maternal glucocorticoids, which in turn affects fetal HPA axis and growth [29, 30]. In line with our previous studies, with nicotine exposure, we found decreased maternal hypothalamic CRH, but increased pituitary ACTH and serum Cort levels; in addition, fetal serum Cort and lung GR levels increased [31, 32]. Previously, it has been shown that the negative feedback from elevated serum Cort and ACTH levels during pregnancy results in inhibited maternal hypothalamic CRH secretion, which normalizes after delivery [63]. It is likely that perinatal smoke/nicotine-induced lung injury in the exposed offspring, at least in part, is causally related to maternal glucocorticoid overexposure. In contrast, EA applied at ST36 throughout pregnancy and lactation results in increased maternal hypothalamic CRH, but decreased pituitary ACTH and serum Cort levels. This effectively restores the maternal HPA axis, avoiding offspring overexposure to maternal glucocorticoids, which normalizes the offspring's serum Cort and lung GR levels, thereby preventing nicotine-induced lung injury.

Our data suggest that maternal EA during pregnancy can have lasting effects on the maternal HPA axis, that is, at least until the end of lactation. Long lasting effects after acupuncture have been demonstrated in other conditions as well [64, 65]. For example, in a rat model, it has been demonstrated that inhibition of morphine withdrawal syndrome lasted 7 days after the end of the treatment [64]. As another example, the beneficial effects of acupuncture anesthesia have been shown to last well into the postoperative recovery period [65]. However, these effects gradually wane, which might explain the lack of beneficial effects in pulmonary function and in PPAR*γ* and serum Cort levels at PND21 following prenatal EA. We also found that although EA applied to ST36 acupoints during lactation modulated maternal HPA axis, it had no apparent effect on offspring rats. It is likely to be due to relatively limited transfer to maternal glucocorticoids via breast milk to offspring. A previous study showed that PPAR*γ* agonists administered during lactation (PND1- PND21) could reverse nicotine-induced lung damage in rat offspring [24]. The contrasting data from that study and our present study are possibly related to the fact that in the previous study the PPAR*γ* agonist was directly administered to rat pups, whereas in the present study, the protective effect was dependent upon transmission of protective factors via breast milk. Overall, our data support that for the optimal benefit of EA at ST36 acupoints against perinatal nicotine-induced lung damage, it needs to be administered both pre- and postnatally.

## 5. Conclusion

In conclusion, in an experimental rat model, maternal EA applied to ST36 acupoints, during both “pre- and postnatal periods,” preserves offspring lung structure and function despite perinatal exposure to nicotine. This effect is accompanied by blockage of PNE-induced changes in HPA axes in both the mother and the offspring, thus preventing offspring exposure to excessive maternal glucocorticoids, which occurs with perinatal nicotine exposure. Maternal EA at ST36, administered exclusively during the “prenatal period,” affords only limited benefit, while its administration exclusively during the “postnatal period” does not afford obvious protection.

## Data Availability

The data used to support the findings of this study are available from the corresponding author (Bo Ji) upon request.
